# Portable dynamic radiography in pulmonary thromboembolism with renal impairment

**DOI:** 10.1093/ehjcr/ytae281

**Published:** 2024-06-11

**Authors:** Takafumi Haraguchi, Junichi Matsumoto, Kojiro Ono, Shigeki Fujitani

**Affiliations:** Department of Radiology, St. Marianna University School of Medicine, Sugao 2-16-1, Miyamae-ku, Kawasaki 216-8511, Japan; Department of Advanced Biomedical Imaging and Informatics, St. Marianna University School of Medicine, Sugao 2-16-1, Miyamae-ku, Kawasaki 216-8511, Japan; Department of Emergency and Critical Care Medicine, St. Marianna University School of Medicine, Sugao 2-16-1, Miyamae-ku, Kawasaki, Japan; Medical Imaging R&D Center, Healthcare Business Headquarters, Konica Minolta, Inc., 2970 Ishikawa-machi, Hachioji-shi, Tokyo, Japan; Department of Emergency and Critical Care Medicine, St. Marianna University School of Medicine, Sugao 2-16-1, Miyamae-ku, Kawasaki, Japan

**Figure:**
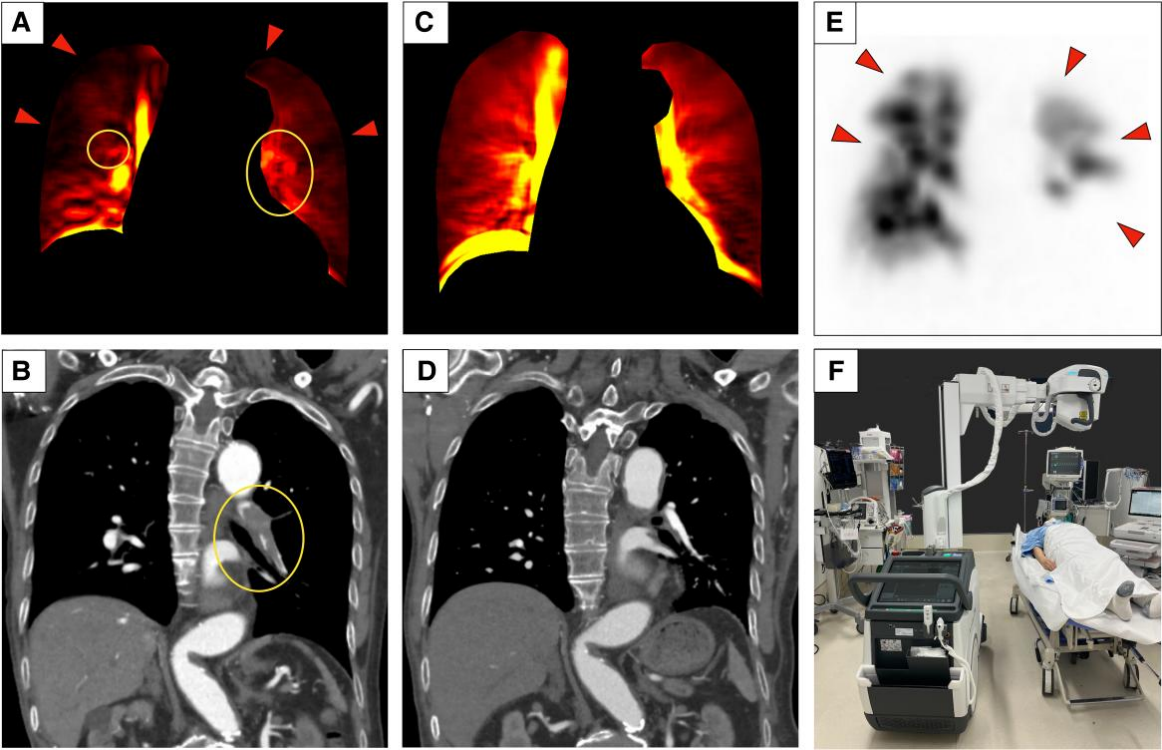


A 79-year-old man with nausea visited the emergency department (ED) by ambulance with decreased oxygen saturation. No abnormalities were noted on computed tomography (CT), performed without contrast due to his renal impairment. Given the elevated D-dimer level, portable dynamic chest radiography (DCR) was performed in the ED using a mobile radiography system (Aero DR TXm01, KONICAMINOLTA, Japan) (*Panel F*) to assess suspected acute pulmonary embolism (PE). One of the modes from DCR imaging analysis (*Panel A*) identified diminished blood flow signals in the proximal pulmonary arteries (yellow circle) and in lung fields (red arrowheads), suggesting PE. On the same day, perfusion scintigraphy confirmed perfusion defects in similar areas of lung fields (*Panel E*, red arrowheads). After renal function improvement, CT pulmonary angiography revealed multiple PEs (*Panel B*, yellow circle) the next day. Post-treatment DCR revealed improved blood flow signals without defects (*Panel C*). Different appearance of pre-treatment image relative to normal one is reminiscent of the ‘melting wings of Icarus’. Follow-up CT pulmonary angiography confirmed disappearance of PEs (*Panel D*).

Dynamic chest radiography is a novel imaging technique providing dynamic X-ray images synchronized with pulmonary ventilation or cardiac pulse. This method provides dynamic images (as does fluoroscopy) derived from analyses of temporal variations in lung-field density, synchronized with pulmonary ventilation or cardiac rhythm. Patients hold their breath enables pulmonary blood flow assessment without requiring contrast agents or radioisotopes. Dynamic chest radiography is also useful for critically ill patients for whom transfer itself poses a risk of deterioration. Portable DCR mobility facilitates bedside use, rendering the technique suitable for emergent use. In imaging findings, the loss of blood flow signals in the proximal pulmonary artery and in lung fields reflects the presence of PE. Although it may not be possible to diagnose due to artefacts caused by body movement, chronic obstructive pulmonary disease, or missing small amounts of PE, the information on pulmonary ventilation and blood flow provided by portable DCR may be invaluable in the management of critically ill patients, as it allows for real-time assessment of lung function and haemodynamics at the bedside without placing a significant burden on the patient.

(*A*) Blood flow signal deficits in the both proximal pulmonary artery (circle) and in both lung fields (arrowheads) in blood flow imaging mode in a portable dynamic chest radiography system. (*B*) A large thrombus (circle) is visible in the left proximal pulmonary artery via computed tomography pulmonary angiography. This figure does not show a thrombus in the right proximal pulmonary artery. (*C*) Portable dynamic chest radiography clearly reveals post-treatment pulmonary flow in both the pulmonary artery and bilateral lung fields. (*D*) Computed tomography pulmonary angiography confirms arterial thrombus disappearance. This figure also does not show the disappearance of a thrombus in the right proximal pulmonary artery. (*E*) Subsequent perfusion single-photon emission computed tomography reveals multiple pulmonary perfusion defects in both lung fields (arrowheads), as dynamic chest radiography did. While the pulmonary artery is normally invisible in perfusion imaging, the absence of the proximal pulmonary artery in dynamic chest radiography (*A*) represents loss of blood flow signal. (*F*) Portable dynamic chest radiography permits dynamic imaging in an emergency department.

## Data Availability

Data generated or analysed during the study are available from the corresponding author by request.

## Notes

**Consent:** Written informed consent was obtained from the patient for publication of this case report and accompanying images.

**Funding:** T.H., J.M., and S.F. received research grant from Konica Minolta to the author's institution.

